# Effect of a multimodal training on the ability of medical students to administer the MMSE: a comparative study

**DOI:** 10.1186/s12909-024-05044-7

**Published:** 2024-02-12

**Authors:** Frédéric Roca, Lucie Lepiller, Camille Keroulle, Doriane Lesage, Kevin Rougette, Philippe Chassagne

**Affiliations:** 1 Department of Geriatric medicine, Rouen University Hospital, 1 rue de Germont Rouen, F 76 000 Normandy, France; 2 INSERM U1096, Normandy University, UNIROUEN, F-76000 Rouen, France

**Keywords:** MMSE, Standardized practical exam, Medical student, Neurocognitive disorders

## Abstract

**Backgrounds:**

The Mini-Mental State Examination (MMSE) is the main screening and follow-up test for neurocognitive disorders. In France, it is often administered by medical students. Conditions allowing to administer the MMSE are strict but not well known by students, leading to mistakes in scoring. Our objectives were to assess the effect of a multimodal training on medical students’ ability to administer the MMSE and to describe their previous training.

**Methods:**

75 medical students between the 4th and 6th year of study were included. Previous MMSE training was assessed by a standardized questionnaire. The teaching material used for our training was the article validating MMSE in French, a video explaining the steps on how to administer the MMSE test, and MMSE’s scoring exercises. The ability to administer the MMSE was assessed by a Standardized practical exam (SPE). Students were self-selected and then assigned in two groups, one benefiting from all the training before SPE, and the other receiving only the article before SPE.

**Results:**

41 students were included in the training group and 34 in the control group. There was no difference between groups regarding previous training. 71% of the students had already administered a MMSE test and only 17% had received specific training. Students considered their previous training as insufficient in most cases. The overall score and scores of each subpart of the SPE were significantly higher in the training group than in the control group (overall score: median [IQR]: 71 [62–78] vs. 52 [41–57], *p* < 0.001). The rate of students able to complete the MMSE was higher in the training group compared to the control (85% vs. 44%, *p* < 0.001). Quality of the training and its usefulness were judged to be good or very good by all participants.

**Conclusions:**

A multimodal training improves the ability of medical students to administer the MMSE.

**Supplementary Information:**

The online version contains supplementary material available at 10.1186/s12909-024-05044-7.

## Key messages

• A multimodal training improves the ability of medical students to administer MMSE.
• After a multimodal training, most of the medical students are suitable to administer MMSE.

**Supplementary Information:**

The online version contains supplementary material available at 10.1186/s12909-024-05044-7.

## Introduction

Major neurocognitive disorders (MND) are common and their prevalence is increasing [1]. MND are underdiagnosed and lead to dependency, repeated hospitalizations and decreased life expectancy [2, 3]. In addition, this diagnosis has an important weight in the decisions to limit care, as MND are frequently associated with limitation of surgical, oncological or intensive care [4–6]. Screening, diagnosis, severity assessment and monitoring of MND are largely based on neuropsychological tests [7]. These neuropsychological tests assess overall cognitive efficiency or are specific to some cognitive domains such as memory, language, praxis or executive functions [7, 8].

Among the tests assessing overall cognitive efficiency, the Mini-Mental State Examination (MMSE) is the most widely used for screening, monitoring and evaluating severity of cognitive disorders, in France and worldwide [9]. It is quick to administer (< 10 min) and validated in many languages. However, it requires strict administration conditions, as well as standardized completion and scoring [10–12]. The MMSE is scored out of 30 points and evaluates temporo-spatial orientation (10 points), learning with an immediate recall of 3 words (3 points), attention by a calculation test (5 points), memory by the delayed recall of the 3 words (3 points), language (8 points) and visuo-constructive praxis (1 point).

This test can be administered by trained neuropsychologists, neurologists, geriatricians, general practitioners but also by other health professionals such as advanced practice nurses or trained nurses [13]. In practice in French hospitals, the MMSE is frequently administered by medical students. However, the scoring of MMSE by medical students is generally misjudged and the administration and scoring instructions are frequently unknown [14]. Moreover, a non-standardized bedside training in hospital wards had a poor inter-rate reliability and does not seem to influence the number of errors made during standardized scoring exercises [14]. In addition, despite short training in the MMSE, scoring by general practitioners is significantly higher than by neuropsychologists, considered as the Gold standard, and only half of trained nurses adequately rate the MMSE on 6 filmed clinical vignettes [13, 15].

Our objective was to evaluate the effect of a standardized multimodal training on the ability of medical students to administer a MMSE test. The secondary objectives were to assess previous training of students to MMSE administration and its impact on our results, and the students’ satisfaction about this training.

## Methods

This single-centre prospective comparative study was carried out in our geriatric department. We included all medical students between the 4th and 6th year, carrying out an internship in the geriatric and post-emergency departments, between July 2021 and February 2022. In France, medical students learn neurology, psychiatry and geriatrics between the 4th and 6th years of study. They spend around 20 h a week in hospital, and are allowed to carry out a clinical examination, including medical questioning, and follow-up under the supervision of a doctor. At our university, students are randomly assigned to different hospital departments for a 2 to 3 months rotation, to learn about different medical specialties.

### Study design (additional Fig. 1)

Year of study, previous training on how to administer a MMSE test, number of MMSEs seen or administered previously, supervision when first administering a MMSE and whether or not students consider their training on how to administer a MMSE sufficient during their medical school were evaluated by questionnaire for each student (Additional Material 1).

Students were self-selected and assigned to 2 groups, according to their availability to receive the different training modules, regardless of their previous training, years of study or affinity:

- A training group which received the standardized multimodal MMSE training, which included the original article validating the MMSE in French and detailing the conditions of administration, a training video explaining the steps on how to administer a MMSE and MMSE scoring exercises session.
- A control group which only received the original article validating the MMSE.

Ability to administer the MMSE was assessed in both groups by a Standardized practical exam (SPE) with a standardized scenario and a simulated patient, in the same way as a one station Objective Structured Clinical Examinations (OSCE), between 2 and 5 days after completion of training or after reading the article, depending on the group.

Students in the control group who did not receive the full training received it after the SPE were completed (Additional Fig. 1).

Students’ satisfaction about the training was evaluated by questionnaire after completion of the training and of the SPE. Quality of the learning materials used, duration, quality of the training and students’ satisfaction with the learning methods used were evaluated. Because the order of modules was different between groups, the evaluation of students’ satisfaction was compared between groups (Additional material 2).

### Training’s description (additional Fig. 2)

The learning material was built for the study and was composed of 4 modules:

- The article validating the MMSE in French and detailing the administration and scoring instructions [10]. This article specifies tolerance or not considering vague responses from patients and number of order’s repetition allowed for examiner so that the test stays standardized. All participants in the study received this article corresponding to the minimum required to administer a MMSE.
- A 25-minute video explaining the steps of the MMSE. It presented a scenario with a neuropsychologist administering the test to a simulated patient with a normal MMSE. This allowed the student to understand in practice the modalities of administering the test, as well as interaction with the patient. Between each step, there was a commented slide describing administration and scoring instructions and common errors made by examiners.
- A one-hour session dedicated to MMSE scoring and error detection in exercise videos. These sessions were carried out in groups of 4 to 8 students face-to-face with a doctor in charge of the study, to promote interactivity. Each session was divided into 2 parts:
  - The first is inspired by Hernandorena et al [14] and consists of 2 videos of role-playing scenario (A and B), used, in our study, for teaching purposes. In these videos a neuropsychologist played her own role and administered a MMSE to a simulated patient. In the video A, the simulated patient responded with 5 standardized errors and students were asked to appropriately score the MMSE. In the video B, the neuropsychologist made 5 administration/scoring errors. Students had to identify these mistakes in a pre-filled MMSE grid. At the end of each exercise a detailed debriefing was made by the doctor in charge of the study.
  - The second was to score the MMSE in four videos recorded during real situations of consultation of our neuropsychologist. Patients’ oral consent was obtained before recording and their faces were blurred to respect their anonymity. A debriefing with the doctor in charge of the study was carried out at the end of each video. The 4 situations in the videos correspond to types of patients frequently encountered in geriatrics (e.g., hypoacusis, Parkinson’s disease, several degrees of cognitive impairment…).
- A SPE to assess students’ ability to administer MMSE. This step had both a teaching and evaluative objective as described below.

### SPE assessment

All the documents related to this SPE are presented in Additional Material 3. In this SPE, the student had to check that the prerequisites conditions (e.g., identity, level of education and ability to read/count of the patient, no severe hearing or visual impairments, no recent psychotropic medication prescription, native French speakers) before administering a MMSE were met and then had to administer it to a simulated patient, played by the doctor in charge of the study, according to a standardized scenario, in a maximum of 10 min. Expected result of the MMSE of the simulated patient was 18/30.

The weighting of the SPE evaluation grid was validated by two geriatricians and a neuropsychologist trained in the administration of the MMSE. Thus, out of a maximum final score of 100 points, 25 points were for the verification of prerequisite administration conditions, 65 points to assess compliance with rules of administration and scoring of the MMSE: 13 points on the orientation part, 10 points on the learning part, 14 points on the calculation part, 3 points on the recall part, 20 points on the language part and 5 points on the praxis part. The last 10 points rated the quality of the relationship between the medical student and the simulated patient, on a scale from 0 to 10, 10 being the best score. The SPE was tested on 5 students before the start of the study. The aim of this test was to train the simulated patient and identify any unexpected responses or reactions of the students in the scenario. After this pre-test, no changes were made to the SPE scenario.

The evaluation was carried out by 2 examiners: the neuropsychologist and a geriatrician of our department trained to administer this test. The neuropsychologist was blind to the student’s group (training or control). In addition to the SPE scoring, the neuropsychologist specified for each student whether he or she was suitable to administer a MMSE.

The SPE also participated in the training of students since each student, regardless of his group, received a personalized debriefing immediately after the SPE by the geriatrician in charge of the study.

### Judgement criteria

The primary outcome was:

1. The comparison of the SPE overall scores between the training and the control groups.

Secondary outcomes were:

2. Comparison of the scores of each subpart of the SPE between these 2 groups.
3. Comparison of the rate of students suitable for the administration of a MMSE between these 2 groups and not misclassifying the severity of the cognitive impairment. A 3-points gap with the expected result of the MMSE of the simulated patient, i.e. out of the 15–21 range, was considered clinically relevant.
4. Comparison of the SPE overall scores between the training and the control groups considering students’ previous training or year of study.
5. Description of the students’ satisfaction about the training.
6. Description of the previous training of students to MMSE administration.

### Statistical analyses

All data were collected and anonymized in a separate Microsoft Excel® file. Analysis was carried out after anonymization. Results are presented as median [IQR 25–75] or absolute value and percent (%).

To assess the primary and secondary outcomes, the 2 groups were compared with a Fisher’s test for qualitative variables and by a Mann Whitney test for quantitative variables. To assess whether previous training impacts our results (secondary outcome 4), SPE overall scores were compared between groups using a linear regression, and the interaction between groups and having seen a MMSE, having administered a MMSE previously or the year of study was evaluated.

A *p*-value < 0.05 was considered significant. Statistics were performed using the R studio 1.4.1106 software.

## Results

### Population characteristics

Seventy-five students were included. Their characteristics are presented in Table 1. Forty-one students were in 6th year, 18 in 5th year, and 16 in 4th year. More than half of students (*n* = 43) had already done an internship in a department used to administrate the MMSE, such as neurology and geriatrics.

**Table 1:** Characteristics of the students before training, overall and according to the group

	Total population *n* = 75	Control group *n* = 34	Training group *n* = 41	*p*-value^1^
Year of medical study				0.231
4th year	16 (21%)	8 (24%)	8 (20%)	
5th year	18 (24%)	5 (15%)	13 (32%)	
6th year	41 (55%)	21 (62%)	20 (49%)	
Previous internship in a ward used to administer the MMSE	43 (57%)	18 (53%)	25 (61%)	0.644
“Expert” ward				0.372
Geriatrics	25 (58%)	12 (67%)	13 (52%)	
Neurology	18 (42%)	6 (33%)	12 (48%)	
Previous MMSE training	13 (17%)	6 (18%)	7 (17%)	> 0.999
Training material used				0.611
Lecture	5 (42%)	2 (33%)	3 (50%)	
Information paper	3 (25%)	1 (17%)	2 (33%)	
Lecture and information paper	4 (33%)	3 (50%)	1 (17%)	
Have previously seen a MMSE	38 (51%)	14 (41%)	24 (59%)	0.171
Number of MMSE seen				0.643
1	13 (35%)	6 (43%)	7 (30%)	
2 to 5	22 (59%)	7 (50%)	15 (65%)	
6 to 10	2 (5%)	1 (7%)	1 (4%)	
Person who showed the MMSE				> 0.999
Medical student	31 (84%)	12 (86%)	19 (83%)	
Geriatrician or neurologist	3 (8%)	1 (7%)	2 (9%)	
Neuropsychologist	3 (8%)	1 (7%)	2 (9%)	
Have previously administered a MMSE	53 (71%)	21 (62%)	32 (78%)	0.144
Number of MMSE administered				0.712
1	11 (21%)	5 (24%)	6 (19%)	
2 to 5	20 (38%)	9 (43%)	11 (34%)	
6 to 10	13 (25%)	5 (24%)	8 (25%)	
> 10	9 (17%)	2 (9.5%)	7 (22%)	
Supervision during the first MMSE				0.832
None	41 (79%)	17 (85%)	24 (75%)	
Medical student	10 (19%)	3 (15%)	7 (22%)	
Geriatrician or neurologist	1 (2%)	0 (0%)	1 (3%)	
Subjective level of training on how to administer a MMSE				0.872
Very insufficient	23 (31%)	12 (35%)	11 (27%)	
Insufficient	33 (44%)	14 (41%)	19 (46%)	
Medium	18 (24%)	8 (24%)	10 (24%)	
Good	1 (1%)	0 (0%)	1 (2%)	

*Results are n (%), ² Fisher exact test. MMSE: Mini-Mental State Examination*

### Status of pre-MMSE training (secondary outcome 6)

Before our training, thirteen students had been trained on how to administer a MMSE. Before administering their first MMSE, half of students (*n* = 38) had seen someone administer one at least once, most often a pear medical student (*n* = 31). Fifty-three students had already administered one or more MMSEs. These students were mostly alone when administering their first MMSE (*n* = 41). Three-quarters of students considered their previous training on how to administer a MMSE to be insufficient or very insufficient (*n* = 56).

Thirty-four students were included in the control group and forty-one in the training group. There was no difference between groups regarding the characteristics of the population (Table 1).

### Comparison of SPE scores

There was no discrepancy in SPE scoring between the neuropsychologist and the geriatrician examiners (Intraclass Correlation Coefficient, ICC = 1) for the parts “verification of prerequisites administration conditions” (25 points) and “compliance with rules of administration and scoring of the MMSE” (65 points), suggesting an excellent inter-observer reproducibility of the scoring grid. The average score of the two examiners was used for the “quality of the doctor-patient relationship” part (10 points), because it was more subjective as demonstrated by a mild ICC at 0.68 (0.50–0.80).

### Outcomes

#### Primary outcome

**Fig. 1:**
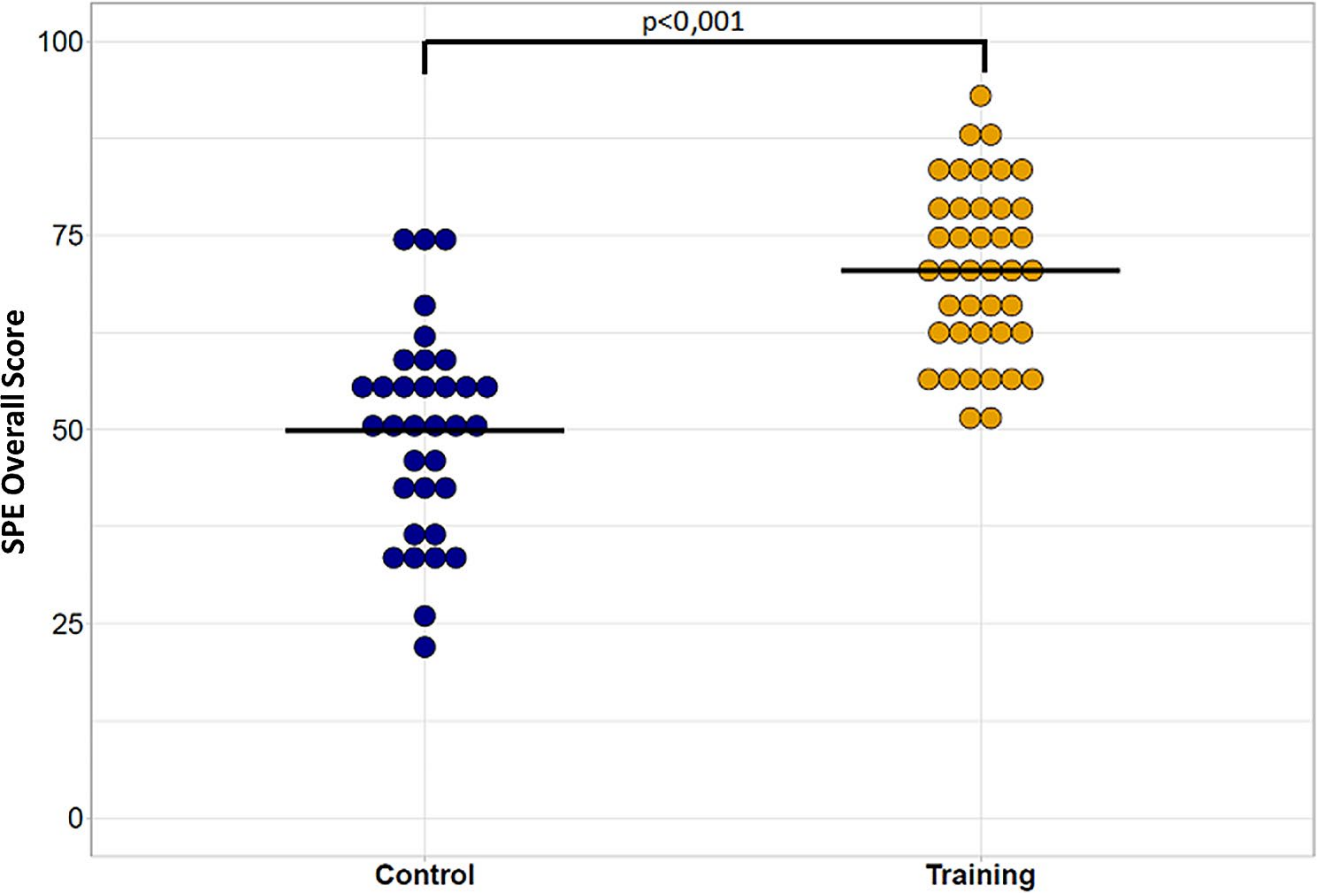
Overall SPE scores in the control and training groups. SPE: Standardized Practical Exam. The horizontal bar is the median. The maximum possible score at the SPE was 100

Students in the training group had a significantly higher overall SPE score than the control group (median [IQR]: 71 [62–78] vs. 52 [41–57], *p* < 0.001) (Fig. 1).

#### Secondary outcome: results for each subpart of the SPE

Students in the training group had significantly higher scores than control for each subpart of the SPE: “prerequisite conditions verification” (median [IQR]: 8 [8–16] vs. 0 [0–4], *p* < 0.001), “administration and scoring the MMSE” (median [IQR]: 55 [51–57] vs. 44 [34–49], *p* < 0.001) or “quality of the doctor-patient relation” (median [IQR]: 7 [7–8] vs. 6 [6–7], *p* = 0.001) (Table 2). The training group had a significantly higher score than the control group for the learning, calculation, recall, and language sub-sections of the “administration and scoring the MMSE” subpart (Table 2).

**Table 2:** Comparison of overall and detailed SPE scores and ability to administer the MMSE between the control and training groups

	Control group, *N* = 34*¹*	Training group, *N* = 41*¹*	*p*-value²
Overall SPE score (/100)	52 [41–57]	71 [62–78]	< 0.001
Prerequisite administration conditions verification (/25)	0 [0–4]	8 [8 − 1]	< 0.001
Doctor-patient relationship (/10)	6 [6–7]	7 [7–8]	0.001
MMSE administration and scoring (/65)	44 [34–49]	55 [51–57]	< 0.001
• Orientation (/13)	11 [9–13]	9 [9–13]	0.532
• Learning (/10)	8 [8–10]	10 [10–10]	< 0.001
• Calculation/Attention (/14)	6 [4–10]	10 [6–10]	< 0.001
• Recall (/3)	3 [2–3]	3 [3–3]	0.012
• Language (/20)	13 [10–15]	20 [18–20]	< 0.001
• Copying (Praxis) (/5)	5 [0–5]	5 [0–5]	0.517
Ability to administer a MMSE	15 (44%)	35 (85%)	< 0.001

^*1*^*Median [IQR] or n (%)*, ^*2*^*Wilcoxon-Mann-Whitney Test or Fisher Exact Test. SPE: Standardized practical exam, MMSE: Mini-Mental State Examination*

*The ability to administer the MMSE was subjectively assessed by the neuropsychologist, blinded to the student’s group*

#### Secondary outcome: ability to administer a MMSE and not misclassify the severity

The rate of students considered as able to administer a MMSE by the neuropsychologist was significantly higher in the training group compared to the control group (85% vs. 44%, *p* < 0.001) (Table 2). Six (17.6%) students in the control group and none in the training group found a MMSE score out of the range of 15 to 21 (*p* = 0.007) and thus could be considered as misclassifying the severity of the patient (Additional Fig. 3).

#### Secondary outcome: impact of previous training/year of study

**Fig. 2:**
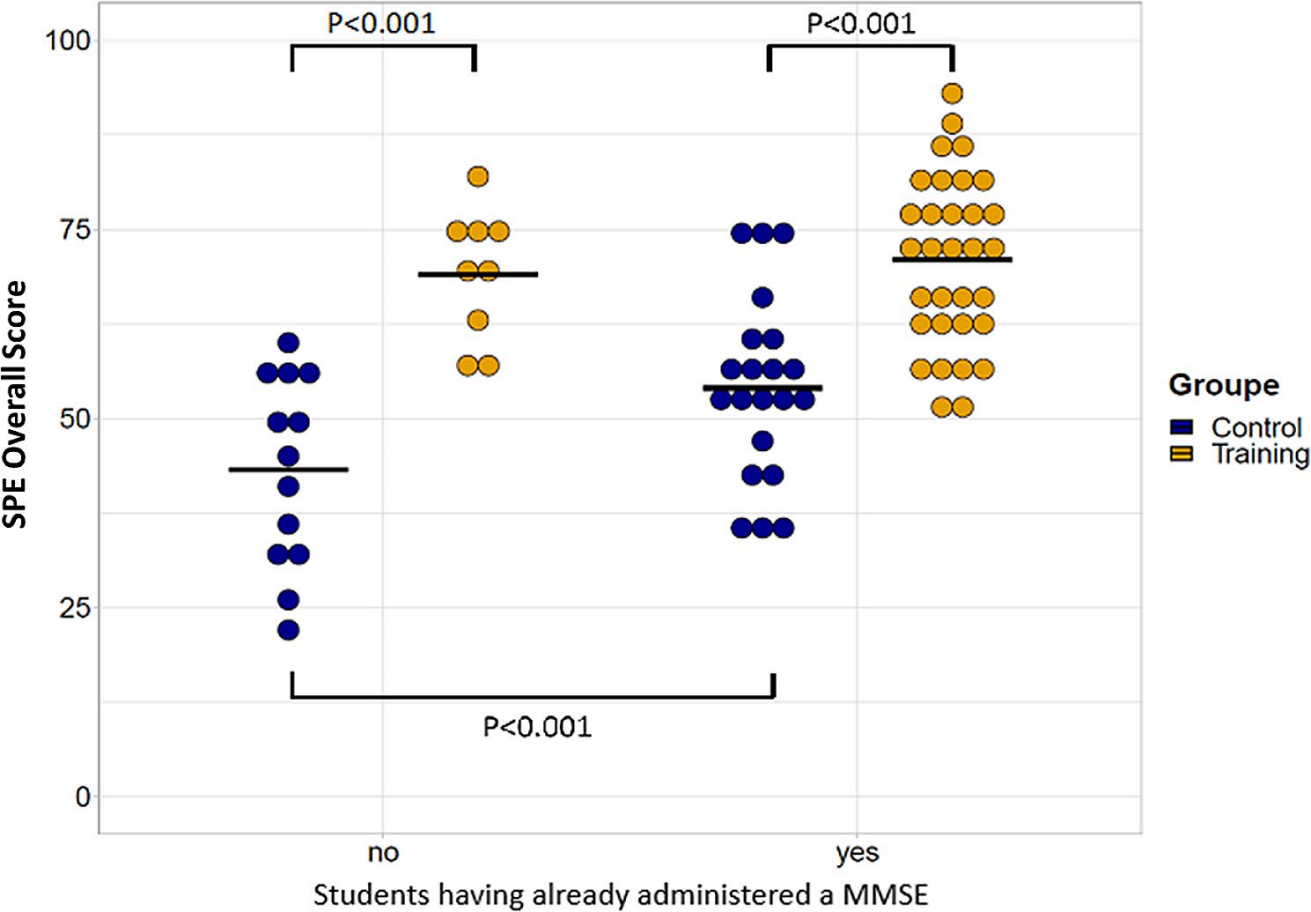
Comparison of overall SPE scores between control and training groups based on whether students had administered or not a MMSE prior to training. SPE: Standardized Practical Exam. The horizontal bar corresponds to the median. The maximum possible score at the SPE was 100

Students who had already administered a MMSE had better overall SPE score than those who had not (ß=11, IC95% [2.8;19], *p* = 0.009) (Fig. 2), but the benefit of the training was similar in these two populations (“groups”*“MMSE administered previously” interaction: ß=-8.9, IC95% [-21;2.8], *p* = 0.130, adjusted R-squared = 0.47). Year of study (4th and 5th year vs. 6th year) or having already seen a MMSE administered did not impact the overall SPE score, (ß=7.2, IC95% [-1.0;16], *p* = 0.089 and ß=4.3, IC95% [-4.0;13], *p* = 0.302 respectively), neither the benefit of the training (*p* = 0.201 and adjusted R-squared = 0.44 for “groups”*“Year of study” interaction and *p* = 0.415 and adjusted R-squared = 0.43 for “groups”*“MMSE seen previously” interaction).

#### Assessment of students’ satisfaction

Three students did not complete the questionnaire of satisfaction. The results of this evaluation are presented in Table 3.

**Table 3:** Satisfaction of the medical students on the training

	Control group, *N* = 32*¹*	Training group, *N* = 40*¹*	*p*-value²
Satisfaction about the article			0.721
Very good	16 (52%)	16 (42%)	
Good	13 (42%)	18 (47%)	
Medium	2 (7%)	4 (11%)	
Satisfaction about the video session			0.833
Very good	15 (47%)	20 (50%)	
Good	13 (41%)	17 (42%)	
Medium	4 (12%)	3 (8%)	
Satisfaction about scoring exercises			0.692
Very good	30 (94%)	36 (90%)	
Good	2 (6%)	4 (10%)	
Satisfaction about SPE			0.071
Very good	23 (72%)	36 (90%)	
Good	9 (28%)	4 (10%)	
Satisfaction about the duration of the video session			0.661
Correct	27 (84%)	32 (80%)	
Long	5 (16%)	6 (15%)	
Too long	0 (0%)	2 (5%)	
Satisfaction about the duration of the scoring exercises session			0.274
Correct	30 (94%)	34 (85%)	
Short	1 (3%)	1 (3%)	
Long	0 (0%)	4 (10%)	
Too long	1 (3%)	1 (3%)	
Most useful module			0.484
Article	2 (6%)	1 (3%)	
Video	0 (0%)	0 (0%)	
Scoring exercises	27 (84%)	32 (80%)	
SPE	3 (9%)	7 (18%)	
Usefulness of the overall training			0.061
Very useful	27 (84%)	25 (62%)	
Useful	5 (16%)	15 (38%)	
Post-training aptitude			> 0.99
Very good	4 (12%)	4 (10%)	
Good	28 (88%)	35 (90%)	
Learning methods used			0.791
Very good	25 (78%)	30 (75%)	
Good	7 (22%)	10 (25%)	

*n (%)*, ^*2*^*Exact Fisher test. SPE: Standardized practical exam*

Quality of the different modules was judged to be good or very good in most cases. All participants rated the quality of the SPE and the scoring exercises as very good or good. Duration of the video training and scoring exercises was considered correct in more than 80% of cases. Students considered the scoring exercise module as the most useful. Usefulness of the training, quality of the teaching and post-training ability to administer a MMSE were considered good or very good by all participants. There was no difference between the two groups regarding all these parameters or according to the year of study.

## Discussion

This study shows for the first time the benefit of a multimodal training on how to administer a MMSE for medical students. We also show that two-thirds of our students have already administered a MMSE, but without prior training or supervision by a senior for most of them. These results confirm an overall impression of geriatricians and neurologists, as well as rare results from the literature, on the insufficient teaching on MMSE tests to French medical students [14]. Neuropsychologists’ learning of the MMSE is based on theoretical teaching as well as practical teaching by tutoring. Neuropsychologists’ ability to administer a MMSE is therefore systematically validated by a qualified neuropsychologist accustomed to the realization of MMSE. Conversely, medical student’s training is incomplete, inhomogeneous and their ability to administered MMSE is rarely validated. However, MMSE requires strict administration conditions that are generally unknown to medical students [14]. Although they have previously administered MMSEs, most of the students consider their training to administer this test to be insufficient. This justifies the implementation of systematic and standardized training during the medical studies course.

Errors in the scoring of a MMSE can occur when instructions on how to administer or score the test are unknown, or by administering a MMSE in unfavourable circumstances (e.g., delirium, major sensorial impairments…). In both situations, consequences are serious since a low MMSE is often interpreted by non-specialized doctors as a synonym for MND. This may wrongly lead to decisions to limit invasive therapies, for example in emergency medicine, intensive care or oncology [4–6].

### Methodological issues

Regarding the methodology used, we provide educational materials that can be used by different speakers. The initial training video can be used on a large scale and in distance learning. Scoring exercise sessions can be carried out by a trained doctor, but also by a neuropsychologist. This teaching material has been developed by 2 doctors who are in the know of the MMSE, as well as by a neuropsychologist, considered as the “Gold standard” in administering the MMSE.

Time required to carry out this training can be considered as short: 25 min of video and 1 h of face-to-face exercise session for up to 8 students. Thus, it is possible to offer a standardized training on how to administer a MMSE to numerous students. We believe that all medical students should be trained on how to administer a MMSE, as screening of neurocognitive disorders is not only carried out by geriatricians or neurologists. General practitioners are at the forefront of this screening, explaining the decision in 2016 of a specific rating for these consultations in France [16, 17]. However, they are not fully trained to carry out these tests [18].

The SPE allows a standardized evaluation of learners, reproducible, with a rating grid y developed by two geriatricians and a neuropsychologist. This type of evaluation seems suitable for the MMSE because of its well-codified conditions of administration and limited interpretation in the scoring grid. Thereby, the inter-observer reproducibility of this SPE rating grid was excellent: there was no difference in scoring between the doctor and the neuropsychologist concerning the parts “prerequisite administering conditions verification” and “MMSE administration and scoring”. At our university, students are used to being assessed by OSCE examinations. Thus, evaluation by a SPE was not a potential bias in our study. In addition, OSCEs are now part of the French national examination for 6th-year medical students, and a station about the ability to administrate a MMSE could be included in this type of examination. In addition, SPE has a teaching objective, allowing students to note their own difficulties in a “real situation” and by the immediate debriefing of the main errors at the end of SPE [19].

In our study, all participants had the article validating MMSE in French, before the SPE. We considered this to be the minimum knowledge required to administer a MMSE. However, in our study as in others [14], students had already administered MMSEs in everyday clinical practice without this minimum knowledge. Thus, our control group has probably a higher knowledge of how to administer a MMSE than that usually provided to students before administering their first MMSE. This may reduce the difference in SPE scores observed between the control and the training groups. In addition, the overall SPE score was better for students who administered a MMSE before our training, suggesting a benefit to a minimal informal bedside training.

### SPE results

Despite this, overall learners’ ability to administer the MMSE was better in the training group, regardless of the administration of MMSEs before training. Each of the 3 components of this SPE was improved by the training, even the “doctor-patient relationship’s quality”. Some studies do not demonstrate the benefit of short MMSE training for general practitioners or nurses. In these studies, students failed to obtain the same final MMSE score as the neuropsychologists [13, 15]. However, the differences between general practitioners and neuropsychologists were slight [15] and, in most cases, patients were not misclassified [13]. This suggests a possible benefit of this training, however as it is not standardized and there is no detailed description of the training, it is difficult to generalize it to all medical students. In addition, the performance of students in MMSE scoring or error detection exercises is poor following informal bedside training on how to administer a MMSE in hospital departments, a usual situation observed in France [14]. Another study shows that when evaluating how to administer a MMSE during a SPE, the MMSE is correctly scored in only 78% of students after a formal training by clinical cases and vignettes, although all students consider themselves able to administer it [20]. However, this study did not precise how scoring was successful. Our results therefore are arguments in favour of a more standardized training, of longer duration, using multiple teaching materials.

### Satisfaction

Satisfaction with our training was good or very good in most cases. Duration of the training was mostly considered appropriate. Quality of materials was highlighted as well as the learning methods used. At the end of this training, all students consider their ability to administer the MMSE to be good or very good, whereas most of them considered it insufficient or very insufficient before. This argues in favour of the generalization of this type of training tool to medical students. In addition, there was no difference between groups in the assessment of training’s quality. This suggests that starting with SPE (control group) was not considered deleterious by students. This can be explained by the interest of having been put in a situation before returning to a more theoretical training.

### Limitations

This study has some limitations. First, SPE scoring is well standardized for the “prerequisites administration conditions” and “MMSE administration” parts, but assessment of the quality of the doctor-patient relationship is more subjective. To limit the risk of bias, we used the average of the scores given by the two examiners. Nor can we be sure what influence the fact that the simulated patient is played by the doctor rather than a real patient has on the evaluation of the relationship. Conversely, the SPE scores were totally correlated between the neuropsychologist and the geriatrician for the “verification” and “compliance” parts, probably because these two examiners work in the same team and were involved in the construction of the scoring grid. This could be a limitation to exporting our SPE to other teams with different examiners.

Second, results might be better in our study than in real life, as students might have revised before taking the SPE more than they would have in real life [19]. However, this bias affects both groups and assessment of students’ ability to administer the MMSE in an unstandardized situation with a real patient, would have been a source of variability. Third, our research protocol could have been improved by using a real simulated patient rather than a doctor, randomizing students into the two groups, keeping the time between training and SPE exactly the same, or assessing MMSE skills before and after training. Fourth, we did not assess the long-term persistence of this training’s benefit. Finally, interpretation of the MMSE was not assessed because it was outside the objectives of the training.

### Perspectives

Perspectives of this work are multiple. First, we want to extend this training to other health care professionals involved in the MMSE administration, such as advanced practice nurses, geriatric nurses and general practitioners training in geriatrics. Second, the dissemination and validation of this training course at national level for French students is already planned. Third, this type of training could be developed for other standardized neuropsychological tests, but also for other geriatric assessment tools such as those dedicated to assessment of dependence, autonomy, or delirium. This could contribute to raising the awareness of future medical doctors about geriatric issues and thus improve the care of elderly patients [21].

## Conclusion

This study demonstrates for the first time that a multimodal training in MMSE improves medical students’ ability to administer this test. Perspectives of this work are numerous, particularly in terms of spreading to other professionals involved in neurocognitive disorders’ screening. Further studies are needed to assess the long-term persistence of learners’ ability to administer a MMSE.

### Electronic supplementary material

Below is the link to the electronic supplementary material.

Supplementary Material 1

## Data Availability

anonymized data are available on reasonable demand to the corresponding author, Dr Frédéric ROCA.

## Abbreviations

MMSE: Mini-Mental State Examination
MND: Major neurocognitive disorders
SPE: Standardized practical exam
