# Supplementary material for: Effect of a multimodal training on the ability of medical students to administer the MMSE: a comparative study
Source: BMC Med Educ. 2024 Feb 12;24:133. doi: 10.1186/s12909-024-05044-7 (PMC10863194; doi:10.1186/s12909-024-05044-7)
Supplement: Supplementary file 1 — Supplementary Material 1 [file 12909_2024_5044_MOESM1_ESM.docx]

# Additional material

**Additional material 1: Pre-MMSE Training Assessment Questionnaire**

Name: First name: Date of birth:

Year of study:

4^th^ 5^th^ 6^th^

Have you ever been in an "expert" hospital ward used to administer the administration of the MMSE (several possible answers)?

Neurology Geriatrics

Have you already received training in how to administer a MMSE?

Yes No

If yes, what type of training (several possible answers)?

- Paper support, description of the administration conditions
- Lectures
- Video lessons
- Scenario (e.g., formative SPE, role-playing games)
- Others, specify: .........

Have you ever seen a MMSE being done?

Yes No

If yes, how many times?

1 1-5 5-10 >10

If yes, by whom? (Only 1 possible answer):

- Medical student
- Neurologist/geriatrician
- Neuropsychologist
- Others, specify: .........

Have you ever administered a MMSE?

Yes No

If yes, how many?

1 1-5 5-10 >10

If yes, when you administered the first MMSE, were you? (Only 1 possible answer):

- Alone with the patient
- With a medical student
- With a neurologist/geriatrician
- With a neuropsychologist
- With a .......... (Specify if other)

Generally, you consider your training in the administration of the MMSE as (1 only possible answer):

- Very insufficient
- Insufficient
- Medium
- Good
- Very good

**Additional material 2: satisfaction questionnaire**

1. **Which training did you receive (circle)?**

SPE then Video Video then SPE

1. **What is your level of satisfaction with the materials used for training?**

|  | Very good | Good | Medium | Bad | Very bad |
| --- | --- | --- | --- | --- | --- |
| Paper article |  |  |  |  |  |
| Initial video training |  |  |  |  |  |
| Scoring exercise |  |  |  |  |  |
| SPE |  |  |  |  |  |

1. **What do you think about the training’s duration?**

|  | Too long | Long | Correct | Short | Too short |
| --- | --- | --- | --- | --- | --- |
| Initial video training |  |  |  |  |  |
| Scoring exercise |  |  |  |  |  |

1. **In your opinion, were the objectives of the training clearly formulated at the beginning of the session?**

Yes No

1. **Which training module seemed most useful to you (only 1 possible answer)?**

Paper article Initial Video training SPE Video scoring exercise

**6- Overall, this training was:**

Very useful Useful Moderately useful Little useful Useless

1. **At the end of this training, you consider your ability to administer a MMSE as:**

Very good Good Medium Insufficient Very insufficient

1. **Overall, how do you evaluate the pedagogy used during this training?**

Very good Good Medium Bad Very bad

Suggestions/free comments:

**Additional material 3: Standardized practical exam Assessment**

# SPE FRAMEWORK

**Reason for consultation:**

You are a geriatrician and you see in consultation your patient of 81 years to whom you wish to administer a MMSE.

**General information:**

Consultation at Charles Nicolle Rouen Hospital, Seine Maritime, Normandy, 4th floor, Tuesday, July 13th, 2021

**Objectives of the station to be measured:**

- Ability to administer a MMSE
- Ability to rate a MMSE
- Knowledge of the prerequisite administration conditions of the MMSE

**Weighting:**

- Verification of prerequisite administration conditions 25 pts
- Administration of the MMSE 65 pts
- Patient-doctor relationship 10 pts

Total 100 pts

**Duration:** 10 minutes maximum

**Equipment to be provided:**

- A pencil and 2 pens,
- A watch,
- A “French” MMSE grid (circle the words "cigare, fleur, porte" and cross the others),
- Sheet with written “Fermez les yeux” (item 28), Sentence (item 29) and Figure (item 30),
- White paper sheets

# Simulated Patient Scenario

**Practice mode:**

You are 81 years old and you are taking a MMSE

**Patient situation and history:**

*"Hello doctor, you told me I would have a memory test today."*

If the doctor asks:

- You do not have a hearing or visual problem
- You do not have medical history except hypertension for 5 years; you do not have depression or an antidepressant treatment
- You have a good moral
- Your mother tongue is French
- You have the baccalaureate, and you were a former manager of two clothing stores
- Drugs: Ramipril 5 mg 1 once a day, Acetaminophen 1 g in case of pain

## The doctor administers the MMSE:

For the question about the full date, answer: "uh… dates, that's what is difficult for me"

Q1: hesitate and then say “2021”

Q2: “In summer”

Q3: “July”

Q4: "It's the 12th... I'm not sure"

- **if the doctor asks again,** say: “the 12^th^ or 13^th^, I think”;
- **if he asks again**, say: “the 13^th^”

Q5: “Today is Monday or Tuesday I think”

- **if the doctor asks to choose:** “Tuesday”

Q6: “Charles Nicolle Hospital”

Q7: “Rouen”

Q8: “76”

- **if the doctor asks to specify**, say: "Seine maritime"

Q9: “The region of Rouen”

- **if the doctor asks again, say:**  "uh… the region of Rouen, Normandy"

Q10: think 2 or 3 seconds and then say: “4^th^ floor, I think”

Q11-13: think for 4 seconds and then repeat: "Cigar, fleur ... and what is the 3rd already?"

- **if the doctor repeats the 3 words,** repeat: "Cigar, fleur, porte"

Q14 -18: hesitate, 2-3 seconds, then say "remove 7?", hesitate 3 seconds, say "I have to remove 7, right?", hesitate 3 seconds, say "it's difficult", **wait for help** **from the doctor** for 10 seconds:

- Then **whether there is help or not** say: "92"
- Then say, "92-7 is 85"
- Then say: "minus 7 is 68"
- Then say, "then 58."
- Then ask, "How much should I withdraw already?"
- **if the doctor gives you the instruction:** say "51", **say nothing otherwise**
- **if the doctor says to continue:** say "I don't know anymore"

To hold the word world upside down: E - D – **M** – O – N

Q19-21: Say: "Cigar", hesitate for 4 seconds, say “I can't remember the other words"

- **If the doctor asks you to think about it, to try to remember**...: say "it doesn't come back to me"
- **If the doctor gives a clue:** for “fleur” (e.g., it is a plant), say: "fleur", for “porte” (e.g., it is used to enter the house), say: "entrée"

Q22: Say "a pencil"

Q23: Try to grab the watch, and whatever the doctor's reaction, hesitate a little and say "a watch"

Q24: Say "Pas de mais, de si, ni de et"

- **If the doctor asks you if you are sure OR if the doctor repeats the phrase:** say "Pas de mais, de si, ni de et”

Q25 -27: After the complete instructions, take the paper with **your left hand,** fold it in half and throw it on the ground

- ***If the doctor waits more than 5 seconds*** *after the first instruction take the paper despite the absence of the complete instructions*

Q28: Read the instruction to close your eyes and then close them

Q29: Write: "The weather is nice"

Q30: Copy the drawing by missing an angle, ask to redo the drawing:

- **If the doctor authorizes you**: redo the drawing without error
- If the doctor does **not authorize you:** do not redo the drawing

**General behaviour:** anxious

**General guidelines:** You must know the scenario perfectly so that you always answer the questions that are asked in the same way.

# Standardized Practical Exam Scoring Grid

**Instructions to the examiner:** The purpose of this station is to assess the ability to administer a MMSE. You must not intervene.

## Verification of the administering conditions: 25 pts

- verifies the identity of the patient 2 pts
- checks the level of study, ability to read or write 8 pts
- checks thymic condition / history of depression /medicines 4 pts
- checks sensory disorders 8 pts
- checks mother tongue 3 pts

## Administering of the MMSE 65 pts

Q4 (*date of the day*) = rated false 3 pts

Q5*(day of the week)* = request to choose 4 pts

Q8 (*department*) = request to specify 4 pts

Q9 (*region*) = rated false 2 pts

Q11-13 (*learning 3 words*) =

- if repetition 8 pts
- if rated 2 words good 2 pts

Q14-18 (*Calculation*) =

- if appropriate help 4 pts
- if rated 1/ (pt given at 92-7) 6 pts
- if said to continue 4 pts

Q19 -21 (*Reminder*) = if rated 1/3 3 pts

Q22 (*pencil*) = if the pencil is put on the table 2 pts

Q23 (*watch*) = if prevents taking the watch or odds 0 3 pts

Q24 (*pseudo sentence*) = if rated 0 4 pts

Q25 - 27 (*sheet*) =

- If gives the paper on the table or in the centre 3 pts
- If gives the complete instructions from the outset 4 pts
- If rated 2/3 4 pts

Q30 (*figure*) = If rated 1 point on the 2nd drawing 5 pts

**Quality of the doctor-patient relationship (circle)**  **10 pts**

*(Asks questions intelligibly, reassures the patient through behaviour...)*

Very bad Excellent

1 2 3 4 5 6 7 8 9 10

**If rating error on item 1-2-3-6-7-10-14-22-28 or 29:** -1 pt /question

**Date:** Ask the 5 questions about the date systematically: -1 pt if not done

# STUDENT’S INSTRUCTIONS

**It's Tuesday, July 13th, 2021**

**You are a geriatrician at Charles Nicolle Hospital in Rouen and you see in a geriatric consultation, (on the 4th floor) your 81-year-old patient to whom you wish to administer a MMSE.**

**YOU MUST:**

Check in a few questions that the administering conditions are met and administer the MMSE.

# Additional Figure 1: Flow-chart and study design

**75 students**

**Control group (n=34)**

**Training group (n=41)**

**Training video session**

**SPE**

**Article validating the MMSE in French**

**MMSE’s scoring exercises**

**Questionnaire for previous training on how to administer a MMSE**

**SPE**

**Assessment of the satisfaction of the students about the training**

*SPE: Standardized Practical Exam, MMSE: Mini-Mental State Examination.*

**Additional figure 2: teaching module**

**Additional figure 3: Scattering of the MMSE score given by students at the SPE in the control and training groups**

*SPE: Standardized Practical Exam. MMSE: Mini-Mental State Examination. Expected MMSE score was 18 (dashed red line). Students scoring MMSE out of the range of clinical relevance (15 – 21, blue lines) are considered as misclassifying the severity of the cognitive impairment.*
